# The protonation state of an evolutionarily conserved histidine modulates domain swapping stability of FoxP1

**DOI:** 10.1038/s41598-019-41819-5

**Published:** 2019-04-01

**Authors:** Exequiel Medina, Pablo Villalobos, Ricardo Coñuecar, César A. Ramírez-Sarmiento, Jorge Babul

**Affiliations:** 1 Departamento de Biología, Facultad de Ciencias, Universidad de Chile, Las Palmeras 3425, Casilla 653, Santiago, 7800003 Chile; 2 Institute for Biological and Medical Engineering, Schools of Engineering, Medicine and Biological Sciences, Pontificia Universidad Católica de Chile, Av. Vicuña Mackenna 4860, Santiago, 7820436 Chile

**Keywords:** Protein folding, Transcription factors, Protein folding

## Abstract

Forkhead box P (FoxP) proteins are members of the versatile Fox transcription factors, which control the timing and expression of multiple genes for eukaryotic cell homeostasis. Compared to other Fox proteins, they can form domain-swapped dimers through their DNA-binding –forkhead– domains, enabling spatial reorganization of distant chromosome elements by tethering two DNA molecules together. Yet, domain swapping stability and DNA binding affinity varies between different FoxP proteins. Experimental evidence suggests that the protonation state of a histidine residue conserved in all Fox proteins is responsible for pH-dependent modulation of these interactions. Here, we explore the consequences of the protonation state of another histidine (H59), only conserved within FoxM/O/P subfamilies, on folding and dimerization of the forkhead domain of human FoxP1. Dimer dissociation kinetics and equilibrium unfolding experiments demonstrate that protonation of H59 leads to destabilization of the domain-swapped dimer due to an increase in free energy difference between the monomeric and transition states. This pH–dependence is abolished when H59 is mutated to alanine. Furthermore, anisotropy measurements and molecular dynamics evidence that H59 has a direct impact in the local stability of helix *H*3. Altogether, our results highlight the relevance of H59 in domain swapping and folding stability of FoxP1.

## Introduction

Proteins are versatile macromolecules with exquisite equilibria between all different intermolecular forces to favour the native state. Understanding of the physical chemical properties of side chain interactions with side^1^ or main chains^2^ from other residues in the folded protein is relevant to describe their role in reaching the native state and also their involvement in several biological processes. This is particularly critical when only a few substitutions are responsible for the functional and biophysical differences between proteins, allowing their diversification throughout protein evolution in nature^3,4^.

One representative case is the extensive forkhead box (Fox) family of transcription factors, distributed among eukaryotic organisms^5^ and responsible for pleiotropic processes during embryonic development and adult cellular homeostasis, such as immune system regulation, glucose metabolism and different tissue functions in humans and other mammals^6,7^. All members of the Fox family are composed by a structurally conserved DNA–binding domain (forkhead) of ~100 amino acid residues that was used to classify these proteins into 19 subfamilies, from A to S^5^. Most Fox members have been described to fold into monomers *in vitro* regardless of the presence of their cognate DNA. However, only the forkhead domain from the P subfamily (FoxP) has been characterized to form domain-swapped dimers in solution both in absence and presence of DNA^8–11^, despite the high sequence identity (~70–80%) with other Fox members. These intriguing aspects motivated the analysis of the structural and functional properties of FoxP proteins and the molecular basis of their unique behaviour.

Canonically, three–dimensional domain swapping is described as a folding-upon-binding mechanism where two or more proteins exchange identical structural segments^12^, breaking native monomeric contacts and establishing them in an intermolecular fashion. The large number of interactions being reformed imposes a critical energy barrier that controls the kinetic and thermodynamic properties of this process, due to the need of monomer unfolding to enable domain swapping^13,14^. Phenotypically, these constraints are evidenced in the form of extremely slow monomer-dimer transitions and low association affinity for most studied proteins.

We previously characterized domain swapping of the forkhead domain of human FoxP1 (hereafter FoxP1), showing that its association equilibrium is reached without needing extensive protein unfolding in ~2 h at 37 °C under low μM protein concentrations^11^. This is nearly 1,000-fold faster and favourable than in other domain swapping proteins^8–11^, thus suggesting that FoxP1 exhibits a different molecular mechanism due to key structure and sequence features. Conversely, specific mutations affecting domain swapping of FoxP proteins are involved in severe diseases such as IPEX syndrome in humans^9,15^. Alas, the limited structural and biophysical characterization of these proteins impedes unravelling the kinetic mechanism of this process and its relationship with their biological role.

**Figure 1:**
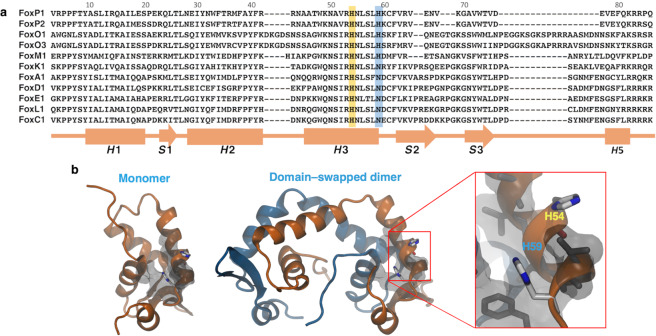
Evolutionary and structural context of H59 in the DNA–binding domain of human FoxP1. (**a)** Sequence alignment of the DNA–binding domain from representative members of the human Fox subfamilies. Histidines highlighted in yellow and blue are the family–conserved and the M/O/P subfamily-specific residues, respectively. A secondary structure topology scheme is shown at the bottom of the sequence alignment. **(b)** Cartoon representation of the structure of the A39P/C61Y monomeric mutant (PDB 2KIU) and the domain–swapped dimer (generated by homology modelling using FoxP2 as template) of the forkhead domain of human FoxP1, showing the position of the two histidine residues (H54 and H59) as sticks. Hydrophobic residues surrounding H54 and H59 at 5 Å are shown in grey using their van der Waals radius.

In this context, Blane and Fanucchi^16^ recently showed the effect of changing the pH around the theoretical pKa of histidine residues on the tertiary structure and DNA binding of the forkhead domain of FoxP2. The pH dependence of these traits was related with protonation changes of a conserved histidine (H54) located in the helix *H*3 of all forkhead domains^7^, due to the inferred role of this residue in protein–DNA binding based on crystallographic^9,10^ and functional^17^ data. However, sequence comparison between several Fox members shows the presence of another histidine (H59) only in the M, O and P subfamilies^7,17^, which could also be relevant for protein function and stability (Fig. 1a). Moreover, the three-dimensional structure of FoxP1 shows that both residues are surrounded by a hydrophobic environment (Fig. 1b), where their interaction ability is limited due to the effective contact radius (~5 Å)^18^. In this scenario, the current structural and biophysical analysis is not sufficient to determine if the consequences observed in FoxP2 were due to changes in the protonation states of the strictly conserved H54 and/or the H59 that is only evolutionarily conserved in the M/O/P subfamilies.

In this study, we explore the impact of each histidine residue in the dimerization and folding stability of the forkhead domain of FoxP1 using both wild type protein (wt) and the H59A mutant. Kinetic analysis of domain swapping shows that only H59 is involved in dimer stability, with its deprotonation significantly increasing the association rate to favour the dimer conformation. This pH-dependent behaviour is also observed in equilibrium unfolding studies, where the global stability of the wt protein, and not the H59A mutant, depends on pH. Moreover, fluorescence anisotropy experiments monitoring local changes in helix *H*3 due to unfolding suggest that its stability is relevant to modulate the dimerization of FoxP1. Finally, molecular dynamics simulations reveal that the protonation state of H59 affects the persistence of a hydrogen bond between its imidazole ring and the carbonyl group of the backbone of residue N55, as well as having a long-range effect in the contact probability of several native interactions on the folding transition state. Altogether, these findings highlight the relevance of H59 in the stability of helix *H*3 and the modulation of the dimerization propensity of FoxP proteins, providing insights of how key residue changes during the evolution of the Fox family of transcription factors aid in sculpting their folding landscape.

## Results

### Protonation state of H59 modulates the domain swapping kinetics of FoxP1

**Figure 2:**
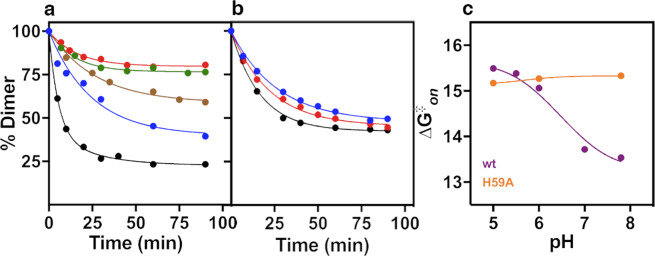
Kinetic measurements of FoxP1 around the pKa of its H59 side chain. Changes in the dimer abundance upon time at different pH values for wt (**a**) and H59A mutant (**b**) after initial incubation of 5 μM of the dimer species. Isolated dimers were incubated at pH 5.0 (black), 5.5 (blue), 6.0 (brown), 7.0 (green) and 7.8 (red) at 37 °C, and dimer population was quantified by SEC experiments. Data was fitted to a single exponential decay. (**c**) Kinetic properties of FoxP1 around the pKa of its H59 side chain. Comparison of ∆G^‡^ (association) of wt and H59A as a function of pH. Values were calculated using the Eyring equation.

To determine if pH changes around the pKa of histidine affect the dimerization and conformational stability of FoxP proteins, we analysed these parameters in a pH range from 5.0 to 7.8 using human FoxP1 as a model. The dependence of monomer-dimer transitions on the (de)protonation of histidine residues was ascertained using size exclusion chromatography (SEC), monitoring the kinetics of dimer dissociation by quantifying monomer and dimer fractions at different times at 37 °C until the reaction reached equilibrium. Results showed a strong effect of pH in the final dimer concentration under equilibrium conditions (Fig. 2a) and also in the kinetics of the monomer-dimer transition (Table 1), with increasing pH favouring the dimer.

**Table 1:** Thermodynamic and kinetics parameters of domain swapping for wt FoxP1 and H59A mutant at 37 °C.

pH	wt				H59A			
	K_d_ (μM)	∆G_d_ (kcal·mol^−1^)	*k*_off_ × 10^–3^ (min^−1^)	*k*_on_ × 10^3^ (M^−1^·min^−1^)	K_d_ (μM)	∆G_d_ (kcal·mol^−1^)	*k*_off_ × 10^–3^ (min^−1^)	*k*_on_ × 10^3^ (M^−1^·min^−1^)
5.0	31.2 ± 0. 3	6.4 ± 0.1	140.9 ± 9.5	3.5 ± 0.7	7.9 ± 0.1	7.2 ± 0.1	59.3 ± 3.5	7.5 ± 0.8
5.5	13.1 ± 0.2	6.9 ± 0.1	40.5 ± 4.0	3.1 ± 0.7	—	—	—	—
6.0	3.7 ± 0.3	7.7 ± 0.1	33.2 ± 3.8	9.0 ± 1.6	5.9 ± 0.1	7.4 ± 0.1	37.7 ± 1.8	6.4 ± 0.3
7.0	0.8 ± 0.1	8.6 ± 0.1	68.1 ± 7.2	80.2 ± 16.4	—	—	—	—
7.8	0.6 ± 0.1	8.8 ± 0.1	63.8 ± 5.1	107.5 ± 13.5	7.5 ± 0.2	7.2 ± 0.2	43.4 ± 2.5	5.8 ± 0.5

This pH dependence can be attributed to protonation changes in either H59 and/or H54 residues. This was corroborated by *in silico* analysis of the monomeric structure of FoxP1 (PDB ID 2KUI) in the H++ server^19^ to estimate the pKa values of titratable residues. We found that only His residues H54 and H59 are effectively titratable in the pH range used herein (data not shown). Thus, to experimentally elucidate the specific role of H59 protonation in the monomer-dimer transition, we generated the H59A mutant to interrupt any possible side chain interaction chemically related with the imidazole ring, and then we performed the same experiment as indicated for the wt protein. In this case, the H59A mutant lost the pH dependence of its monomer-dimer equilibrium, and also the kinetic decay was scarcely affected by pH changes when compared to the wt protein (Fig. 2b and Table 1).

In order to quantify how the protonation state of these residues affects the stability of the domain-swapped dimer, we calculated the equilibrium dissociation constant (K_d_) at each pH. For the wt protein, K_d_ values were strongly affected by pH, ranging from 31.2 ± 0.3 to 0.6 ± 0.1 μM when pH is increased from 5.0 to 7.8 (Table 1). The free energy differences between monomer and dimer (∆G_d_) as a function of pH calculated from K_d_ evidence a dramatic transition between pH 5.5 and 7.0. These results suggest that changes in the protonation state of His residues occurring in this pH range modulate domain swapping stability by ~2.4 kcal·mol^−1^, with an increase in K_d_ occurring at pH values in which His residues are expected to be protonated. In contrast, the H59A mutant shows a steady ∆G_d_ of ~7.3 kcal·mol^−1^ (Table 1), with an increased dimer stability at pH values in which His residues are expected to be protonated (pH < 7) but a decreased stability when the His residue is expected to be deprotonated (pH > 7), when compared to the wt protein. These results suggest that the pH dependence of domain swapping with thermodynamics in the wt protein is due to the equilibrium between the protonated and deprotonated forms of H59 instead of H54, concluding that the latter is mostly involved in protein-DNA interactions^16^ and does not participate, at least directly, in protein-protein interactions.

This pH dependence of the monomer-dimer equilibrium in FoxP1 could emerge from changes on either the association (*k*_on_) or dissociation (*k*_off_) rate constants, having a critical effect in the energetic barrier between monomer and dimer. Therefore, we fitted the dissociation kinetics of the wt and the H59A mutant proteins to single exponential decays to determine *k*_off_, and then *k*_on_ was derived from the relationship between the equilibrium and kinetic dissociation constants as in Eq. 1 (see Methods). Data obtained from these analyses (Table 1) indicates that the main effect of pH is on *k*_on_, showing a dramatic increase of ~50-fold when FoxP1 is changed from acidic to basic conditions with respect to the pKa of the imidazole ring.

To further elucidate the effect of H59 in domain swapping, we used this kinetic information to calculate the free energy changes in association (∆G^‡^_on_) and dissociation (∆G^‡^_off_) using the Eyring approximation (Fig. 2c). First, we observed that the ∆G^‡^_on_ and ∆G^‡^_off_ values described herein were significantly lower than those previously reported for other domain swapping models, such as the C–terminal domain of SARS virus protease (M_pro_–C)^20^, despite the similar K_d_ (Fig. 2c and Supplementary Fig. S1). These results highlight the low kinetic barrier for domain swapping in FoxP1. Secondly, ∆G^‡^_on_ decreased by ~2.5 kcal·mol^−1^ upon increasing the pH above 6.0. Altogether, these findings strongly suggest that deprotonation of H59 in the transition state favours domain swapping of FoxP1.

As expected, H59A shows only a marginal decrease in *k*_on_ and no significant changes in ∆G^‡^_on_ and ∆G^‡^_off_ (Table 1, Fig. 2c and Supplementary Fig. S1), further supporting the specific relevance of H59 in modulating the height of the energetic barrier for domain swapping. Taking into advantage the solely presence of H54 in this mutant, we used the Wyman–Tanford relationship (Eq. 2, see Methods), which relates the pH-dependent free energy changes with the pKa of any residue involved in the dimerization process^21^, to determine the pKa changes of H54 and H59 in the dimerization pathway. Given that no significant changes in ∆G^‡^_on_ were observed for the H59A mutant upon changing the pH, the behaviour of H54 was explained by a pKa of ~5.3. Using this value as a fixed parameter for the analysis of the ∆G^‡^_on_ values obtained for the wt protein, we obtained the pKa of the H59 residue. Our values estimate a decrease of the pKa of this residue by approximately 1.8 units upon reaching the transition state for domain swapping (calculated pKa (M) = 7.3; pKa (‡) = 5.5; pKa (D) = 5.5).

### Protonation state of H59 is involved in global folding stability

Once the role of H59 in the dimerization of FoxP1 was determined, we evaluated the potential involvement of this residue in conformational stability. For these analyses, we compared the equilibrium folding of wt FoxP1 and the H59A mutant, since both folding and association events concurrently take place during its domain swapping^11^.

**Figure 3:**
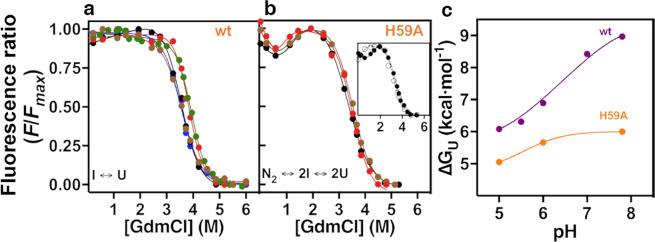
Equilibrium unfolding experiments of wt FoxP1 and H59A mutant. Protein unfolding as a function of denaturant (GdmCl) was monitored using intrinsic fluorescence for wt (**a**) and H59A (**b**) at a total protein concentration of 15 μM. (**a**) wt FoxP1 was incubated with different denaturant concentrations at pH 5.0 (black), 5.5 (blue), 6.0 (brown), 7.0 (green) and 7.8 (red), and the data obtained were fitted to a two-state I $\leftrightarrows$ U monomer unfolding. (**b**) The same unfolding experiment was performed with the H59A mutant at pH 5.0, 6.0 and 7.8, using the same colour pattern as for wt protein. Data was fitted to a three-state N_2_ $\leftrightarrows$ 2I $\leftrightarrows$ 2U unfolding mechanism. ***Inset****.* Unfolding curves of the H59A mutant at pH 5.0 using 3 (white circles) or 15 μM (black circles) of total protein concentration. (**c**) ∆G_u_ dependence with pH for wt and the H59A mutant. Free energy values for intermediate unfolding (I $\leftrightarrows$ U) obtained were fitted to Wyman-Tanford relationship (Eq. 2) to calculate the pKa changes between the intermediate and unfolded states.

In order to analyse the equilibrium unfolding mechanism, we used the intrinsic fluorescence of tryptophan residues W33, W48 and W73 to monitor changes in tertiary structure as a function of the GdmCl concentration at different pH values (Fig. 3). Although we previously showed that the unfolding mechanism of FoxP1 is characterized by the presence of a monomeric intermediate, as ascertained by circular dichroism experiments (N_2_ $\leftrightarrows$ 2I $\leftrightarrows$ 2U)^11^, changes in tryptophan fluorescence for all pH values monitor only one transition. This transition occurs within the same range of denaturant concentrations in which the intermediate, seen by circular dichroism, is in equilibrium with the unfolded state (I $\leftrightarrows$ U)^11^ (Fig. 3a). The absence of the N_2_ $\leftrightarrows$ 2I transition could be explained by (1) masking of this transition due to the heterogeneous fluorescence signal of the multiple tryptophan residues of FoxP1; and/or (2) the positions of the tryptophan residues are insensitive to dimer dissociation. For these reasons, unfolding data from wt FoxP1 was fitted to a two-state mechanism (I $\leftrightarrows$ U).

Our equilibrium unfolding experiments revealed that the unfolding free energy (∆G_U_) of wt FoxP1 depends on pH in a similar fashion to what we observed for the domain swapping process (Table 2), with ∆G_U_ varying from 6.1 to 8.9 kcal·mol^−1^ (Fig. 3c). Although the data were analysed in terms of a two–state mechanism, we observed an increase in the *m*-value of unfolding in the same pH range as the dissociation measurements (Table 2); when pH is changed from pH 5.0 to 7.8, the *m*–value changed from 1.8 ± 0.1 to 2.2 ± 0.1 kcal·mol^−1^·M^−1^. This change accounts for a relative increase of ~29% in the cooperativity of the I $\leftrightarrows$ U transition.

**Table 2:** Thermodynamic parameters of conformational stability of the wild-type and H59A mutant at 37 °C.

pH	Wild-type		H59A					
	∆G_u_^*^	*m–*value^†^	∆G_1_^*^	*m*_1_^†^	∆G_2_^*^	*m*_2_^†^	∆G_U_^*^	*m*^†^
5.0	6.1 ± 0.5	1.7 ± 0.1	7.9 ± 0.9	3.0 ± 0.1	5.0 ± 0.4	1.4 ± 0.1	17.9	5.8
5.5	6.3 ± 0.2	1.8 ± 0.1	—	—	—	—	—	—
6.0	6.9 ± 0.5	2.0 ± 0.1	8.6 ± 0.9	3.2 ± 0.2	5.6 ± 0.7	1.6 ± 0.2	19.8	6.4
7.0	8.4 ± 0.4	2.2 ± 0.1	—	—	—	—	—	—
7.8	8.9 ± 0.4	2.3 ± 0.1	8.2 ± 1.2	3.0 ± 0.1	6.0 ± 1.0	1.8 ± 0.1	20.2	6.6

^*^Expressed as kcal·mol^−1^.

^†^Expressed as kcal·mol^−1^·M^−1^.

This pH-dependence of the changes in *m*-value in apparent two–state systems has been previously rationalized to understand the unfolding features of staphylococcal nuclease, ribonuclease A and T1, and α–lactoalbumin. From these works, it was determined that increases in *m*-values concomitant with increases in pH can be explained by an increase in cooperativity due the destabilization of the I and U states relative to the N state^22^. Examination of the *m*-values obtained for the wt protein show this behaviour: upon lowering the pH, the apparent unfolding cooperativity of the I $\leftrightarrows$ U decreases. This could be an indicator of the presence of the previous characterized intermediate^11^, which would be stabilized at low pH.

In contrast to the wt protein, the equilibrium unfolding of the H59A mutant followed by intrinsic fluorescence showed two clear transitions (Fig. 3b); the first, observed between 1–2 M of denaturant, whereas the second transition matches the I $\leftrightarrows$ U transition described for the wt protein (Fig. 3a). It is worth noting that the first transition is in good agreement with the N_2_ $\leftrightarrows$ 2I transition ascertained for wt FoxP1 using circular dichroism^11^. This is consistent with the fact that a large proportion of dimer is expected at a protein concentration of 15 μM, based on the K_d_ values throughout all the pH range analysed. In order to corroborate that the unfolding behavior is explained by a three-state N_2_ $\leftrightarrows$ 2I $\leftrightarrows$ 2U mechanism as in the case of the wt protein when monitored by circular dichroism, we performed an unfolding experiment at pH 5.0 using 3 μM of total protein (Fig. 3b *inset*), 5-fold lower than the previous experiment. Data obtained at this protein concentration showed a clear shift in the denaturant concentration midpoint (Cm) of the first transition (Cm[3 μM] = 0.6 M; Cm[15 μM] = 1.3 M), consistent with a three-state N_2_ $\leftrightarrows$ 2I $\leftrightarrows$ 2U mechanism. These results suggest that changes in the local environment of H59 are possibly affecting the quantum yield of any of the three tryptophan residues in FoxP1, thus turning intrinsic fluorescence into a good observable property to monitor the local unfolding and dissociation events preceding the formation of the monomeric intermediate.

**Figure 4:**
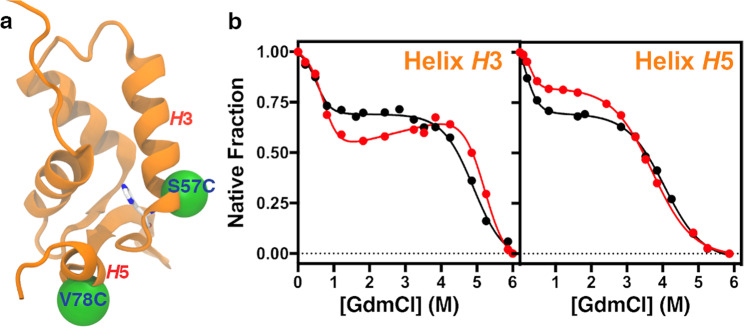
Equilibrium unfolding experiments of helices *H*3 (S57C) and *H*5 (V78C). (**a**) Structural positions of the attached fluorophores onto the specific mutants S57C (*H*3) and V78C (*H*5). (**b**) Unfolding experiments as a function of the GdmCl concentration ascertained by measuring the changes in fluorescence anisotropy for S57C and V78C. Experiments were carried out using 0.1 μM of labelled protein, incubated with different denaturant concentrations at pH 5.0 (black) and 7.8 (red). Data obtained in all experiments were fitted to a three-state monomer unfolding.

Data derived from unfolding curves were used to calculate the energetic parameters of the H59A mutant (Table 2), corroborating the relative insensitivity of *m–*values and ∆G_1_ from the N_2_ $\leftrightarrows$ 2I transition as in the kinetic analysis of the dimer–monomer reaction (Fig. 2 and Table 1). Despite that the N_2_ $\leftrightarrows$ 2I event is largely unaffected by pH (Fig. 3b and Table 2), the protonation change in the remaining H54 residue has an implicit energetic cost of ~1 kcal·mol^−1^ on ∆G_2_ of the intermediate unfolding event (I $\leftrightarrows$ U), which is moderate when compared to wt FoxP1 (Fig. 4c).

As previously done for the kinetic measurements with the H59A mutant and wt protein, we also analysed the ∆G_U_ dependence of both proteins on pH changes using the Wyman–Tanford approach. With this analysis, we attempted to evaluate the possible changes in pKa values of the histidine(s) during unfolding of FoxP1 (Eq. 2), indicating differential protonation states between intermediate and unfolded conformations (Fig. 3c). The pKa values calculated for the H59A mutant showed only a moderate increase in the unfolded state (pKa_I_ = 5.3; pKa_U_ = 5.9), indicating that this residue is scarcely altered in the unfolded ensemble of the protein. However, pKa values derived from the wt protein using the pKa values from H54 as fixed parameters showed a dramatic change in the protonation behaviour of H59 residue (pKa_I_ = 5.7; pKa_U_ = 7.7).

Altogether, these results indicate that (i) protonation changes in residue H54 are mostly related to modulation of protein-DNA interactions^9,10,16^; and (ii) H59 is preferentially deprotonated in order to maintain a native-like environment and increase conformational stability.

### Protonation changes of H59 have a localized impact in folding stability

Given that the protonation/deprotonation equilibrium of H59 is highly involved in the domain swapping stability of FoxP1, we analysed their local effects by creating two single–cysteine mutants within helix *H*3 (S57C) and *H*5 (V78C), both in the vicinity of H59, to attach an extrinsic fluorescent dye sensible to local structural changes (Oregon Green 488; OG488). These regions were chosen since they are exchanged with the adjacent monomer during domain swapping of FoxP1 (Fig. 4a).

Freshly labelled, SEC-purified S57C and V78C proteins were used for equilibrium unfolding experiments in which local changes were monitored using fluorescence anisotropy at pH 5.0 and 7.8. In these conditions, the imidazole rings of H59 and H54 are expected to be fully protonated or deprotonated, respectively, based on the calculated pKa values. Moreover, we used protein concentrations of 0.1 μM, in which the monomer fraction is the most populated state, to focus our measurements on folding stability effects. Results for both proteins clearly indicate two transitions at pH 5.0 and 7.8 (Fig. 4b), thus being consistent with a three–state N $\leftrightarrows$ I $\leftrightarrows$ U monomer unfolding mechanism. The first transition falls between 0.2–1 M of denaturant for both mutants, but the second transition occurs between 4–6 M of denaturant for S57C and 3–5 M for V78C (Fig. 4b), suggesting that unfolding of helix *H*5 precedes that of *H*3.

By fitting the fluorescence anisotropy data from both proteins to a three-state N $\leftrightarrows$ I $\leftrightarrows$ U monomer unfolding mechanism, we determined that the first transition (N $\leftrightarrows$ I) shows no significant differences in its ∆G_1_ value regardless of the pH. Moreover, the changes in stability of helices *H*3 and *H*5 during the N $\leftrightarrows$ I transition, with ∆G_1_ values ranging between 0.6–2 kcal·mol^−1^ (Table 3), are low when compared to the global value obtained for the wt and H59A mutant in conditions where the dimer is the most populated species (Table 2). Nevertheless, a significant increase in *m-*value for helix *H*5 (Table 3) is observed when H54 and H59 are deprotonated (pH 7.8) whereas helix *H*3 is unaffected, suggesting different impacts of the changes in the protonation state of H59 on the local cooperativity of the N $\leftrightarrows$ I transition.

**Table 3:** Thermodynamic parameters of local stability of S57C and V78C mutants at 37 °C.

pH	S57C				V78C			
	∆G_1_^*^	*m*_1_^†^	∆G_2_^*^	*m*_2_^†^	∆G_1_^*^	*m*_1_^†^	∆G_2_^*^	*m*_2_^†^
5.0	2.0 ± 0.5	3.2 ± 1.1	6.9 ± 0.8	1.4 ± 0.2	1.4 ± 0.3	3.1 ± 0.5	4.2 ± 0.2	1.1 ± 0.1
7.8	1.6 ± 0.4	2.7 ± 0.6	10.4 ± 1.1	2.0 ± 0.4	0.6 ± 0.1	5.0 ± 0.9	4.7 ± 0.5	1.1 ± 0.1

^*^Expressed as kcal·mol^−1^.

^†^Expressed as kcal·mol^−1^·M^−1^.

The second transition (I $\leftrightarrows$ U) reveals relevant energetic differences between helix *H*3 and *H*5. First, the free energy change of unfolding of the intermediate state (∆G_2_) monitored from helix *H*3 at pH 5.0 is clearly higher than for helix *H*5 by 2 kcal·mol^−1^. These differences highlight different stabilities that depend on the structural context of both helices. Concomitantly, deprotonation of H59 (pH 7.8) leads to a significant increase in stability of around 3.5 kcal·mol^−1^ of helix *H*3 and not *H*5, which is similar to the energetic change (∆∆G_U_ [pH 7.8–pH 5.0]) obtained from global unfolding analysis with the wt protein. These results suggest that H59 is involved in the pH-dependent stabilization of helix *H*3.

### Protonation of H59 alters hydrogen bond networks and long-range interactions in FoxP1

The experimental evidence above indicates the relevance of the protonation state of H59 in maintaining the folding stability and cooperativity of the region comprising helix *H*3 in FoxP1. These results can be attributed to the ability of the imidazole ring to establish hydrogen bonds, which are lost in the protonated form^23^.

**Figure 5:**
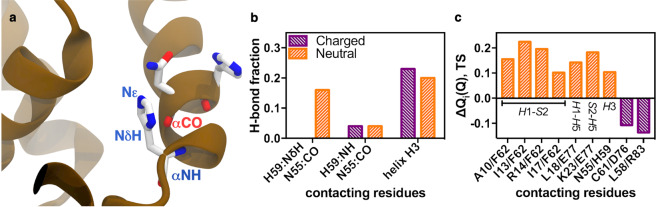
Hydrogen bond and contact probability changes due to protonation of H59 in MD simulations. (**a**) Structural representation of helix *H*3, depicting H59 and N55 interaction, and showing the side chain nitrogen atoms (δ and ε) of the H59 around carbonyl group (CO) of N55. (**b**) Side chain backbone and backbone-backbone hydrogen bond quantifications between charged (purple) or neutral (orange) H59 and N55 from helix *H*3, as well as total hydrogen bonds for helix *H*3. (**c**) Differences in contact probability on the folding TS for monomeric FoxP1 between residues from helices *H*1, *H*3 and *H*5 and strand *S*2. Changes towards higher contact probability for the deprotonated H59 are shown in orange, whereas changes favouring the protonated state are shown in purple.

In order to decipher the specific H-bonds gained or lost due to changes in the protonation state of H59, we performed all–atom molecular dynamics (MD) simulations using either protonated (HSP) or deprotonated (HSD) H59 (Supplementary Fig. S2). It is worth noting that, although the H++ server estimates that both H54 and H59 are titratable under the experimentally evaluated pH range herein, we only modified the charge state of H59 due to the negligible impact of H54 in protein folding and stability on FoxP1 (Fig. 3) and its side chain location on the protein surface (Fig. 1) to interact with the DNA^16^. Interestingly, the major differences observed between both simulations are located within helix *H*3. The introduction of a protonated histidine introduces a perturbation in the N-terminal region of this secondary structure element due to changes in hydrogen bonds between H59 and N55 from helix *H*3 (Fig. 5). Quantification of hydrogen bonds using a distance (donor-acceptor distance ≤ 3.0 Å) and angle (≤20°) criterion show that the imidazole ring of H59 and the backbone carbonyl group of N55 interact during ~20% of the simulation only in the deprotonated state, whereas distances between the backbone amide carbonyl groups of the same residues did not show relevant differences upon deprotonation (Fig. 5b). This change causes an increase of hydrogen bonding on helix *H*3 (Fig. 5b). These results suggest that the interaction of the side chain of deprotonated histidine and the carbonyl group of asparagine is key to stabilize the N-terminal region of helix *H*3.

Given that typical explicit-solvent MD do not regularly sample protein folding transitions, except in cases where special-purpose supercomputers for MD simulations are employed on proteins that fold below the millisecond timescale^24,25^, and that charges also have a strong effect on protein folding and stability due to non-native interactions^26^, we performed folding simulations using coarse-grained structure-based models^27^ augmented by the use of charges^26^ in all E, D, K and R residues, while also manipulating the charge state of H59. Simulations were run at several temperatures above and below T_F_ allowed sampling several (>100) folding-unfolding transitions, thus allowing to integrate the data and calculate the folding landscape of monomeric FoxP1 (Supplementary Fig. S3). From these simulations, we observed a slight increase in T_F_ upon deprotonation of H59. Then, we used folding equilibrium simulations at T_F_ of the protonated FoxP1 to determine which native contacts were responsible for this change in stability and whether these changes occurred in the transition state (TS) or the native state. As shown in Fig. 5c, 7 native interactions showed a significant (>10%) increase in contact probability in the TS upon deprotonation. Moreover, these changes in contact probability were not present in the native state (data not shown). Most of the interactions involved regions near H59 (Supplementary Fig. S4), including four interactions between helix *H*1 and strand *S*2 (residue F62), and the native contact between H59 and N55 (Fig. 5c). The latter is in line with the results from explicit-solvent MD simulations for monomeric FoxP1 (Fig. 5b).

Altogether, these computational results aid in understanding how the deprotonation of H59 leads to local stabilization of helix *H*3 and global stabilization of the transition state and native-like intermediate state of FoxP1.

## Discussion

In this work, the dependence of FoxP1 folding and dimerization on the protonation state of evolutionary histidines were analysed using both wt and the H59A mutant. Dissociation measurements indicated that protonation of the FoxM/O/P-exclusive H59, and not the family-conserved H54, imposes an energetic modulation of ~2.5 kcal·mol^−1^ in the monomer–dimer equilibrium (Fig. 2). This effect is observed only in the association process (Table 1), indicating that monomer and dimer are not equally sensitive to the protonation of the H59 residue. Despite this behaviour, the energetic barrier calculated between monomer and dimer is dramatically lower than in other domain swapping models^20,28,29^, which explains why the dimerization equilibrium of FoxP proteins is reached in hours under physiological conditions^11,30,31^.

In the same line, global unfolding experiments also indicated the direct relationship between protonation equilibrium of residue H59 and folding stability, observing the presence of an intermediate whose population is increased when this residue is protonated. H59A mutant only showed a change in ~1 kcal·mol^−1^ in these conditions (Fig. 3), corroborating both the relevance of H59 in the structural properties of FoxP1 and the lesser impact of the chemical properties of H54 in this context. Anisotropy measurements indicated, firstly, differences in local stability between helices *H*3 and *H*5 and, secondly, that the stability of helix *H*3 is affected by protonation changes of H59 (Fig. 4). These results strongly suggest that local structure changes modulated by changes in the protonation of histidines have an impact in global protein stability of FoxP1. This is not surprising, considering the relevance of the imidazole ring in modulating a myriad of aspects of protein folding^23,32–35^ and function^23,36^ through hydrogen bond (donor and acceptor), cation–π and cation–aromatic interactions^23,37,38^.

Our results are further explained by MD simulations that ascertain the protonation state of H59, suggesting that its stabilization effect is mediated by hydrogen bonds between the imidazole ring and the carbonyl group of the backbone of N55 (Fig. 5b). Simulations performed with the H59N mutant of FoxP1 (conserved in most of Fox members) corroborated the relevance of the persistence of this interaction (data not shown). Moreover, the protonation state of H59 has an impact in increasing the stability of specific residue–residue contacts within the folding transition state of FoxP1, as shown by folding simulations using SBM models (Fig. 5c).

The significance of the single–point change from asparagine to histidine observed only in specific Fox subfamilies seems to be non-trivial when physicochemical properties between these residues are compared in terms of protein stability. Both residues possess the ability to interact with others as hydrogen bond donors and/or acceptors^39^. However, only histidine is affected by pH in a physiological range due to its pKa, acting as a molecular switch that modulates the microenvironment of several active sites and relevant regions of proteins^23,36,40^. This mutation could then represent an ancestral and specific physicochemical change to locally destabilize the monomer structure due to microenvironment changes, promoting the loosening of local regions and the association via domain–swapping, which in turn allows stabilizing these elements in an intermolecular context.

A possible explanation of our results is thus related with the evolutionary context of Fox proteins. We postulate that the presence of this evolutionary histidine, which is only conserved in M, O and P subfamilies, is one of the many accumulative transitions in the pathway towards the emergence of the dimerization ability of FoxP proteins, summative to the prerequisite of alanine (FoxP members) instead of proline (most Fox proteins) in the hinge loop region to enable domain swapping^11^.

## Methods

### Protein expression and purification

Codon-optimized DNA sequence encoding the forkhead domain of human FoxP1 and its mutants were cloned into a modified pET-28a vector, containing a His6-tag, a TEV cleavage site and an S-tag sequence in the 5’ end of the gene. Amino acid residues are numbered according to the sequence numbering in the deposited structure of the A39P/C61Y mutant of the forkhead domain of human FoxP1 (PDB ID 2KIU)^8^. Single-point mutants were generated by PCR using the QuickChange Site-directed Mutagenesis kit (Stratagene, La Jolla, CA, USA). Proteins were purified as described by Medina *et al*.^11^ and dialysed into standard buffer (20 mM HEPES pH 7.8, 150 mM NaCl, 2 mM β–mercaptoethanol) prior to each experiment, unless otherwise indicated.

### Size-exclusion chromatography (SEC)

Monomer and dimer fractions of wt FoxP1 and H59A mutant were quantified and isolated on a Waters Breeze HPLC system (Waters Corporation, Milford, MA, USA) using a Superdex 75 10/30 column (GE Healthcare Biosciences, Pittsburgh, PA, USA) as described in Medina *et al*.^11^. Briefly, the column was equilibrated with 45 ml of mobile phase (standard buffer) at room temperature before SEC experiments. When required, isolated fractions were kept on ice until their use in subsequent experiments.

### Dissociation kinetics

Isolated dimer populations of wt FoxP1 and H59A mutant were diluted into standard buffer at different pH values (5.0, 5.5, 6.0, 7.0 and 7.8) to a final protein concentration of 5 μM and further incubated for 2 h at 37 °C in a water bath. Aliquots after different times of incubation were analysed using SEC, and dimer and monomer concentrations were quantified using the area under the curve for each species compared to the total area of the corresponding elution profile.

### Equilibrium unfolding experiments

Unfolding experiments were performed in buffer at different pH values (5.0, 5.5, 6.0, 7.0 and 7.8) supplemented with GdmCl (molecular biology grade; Thermo Fischer Scientific, Waltham, MA) at concentrations ranging from 0 to 6 M. For all unfolding experiments, the proteins were exposed to different GdmCl concentrations for at least 2 h at 37 °C using a water bath. Unfolding curves were obtained using 3 or 15 μM of protein in terms of monomer concentration. For extrinsically labelled proteins, unfolding experiments were performed using 0.1 μM of protein in terms of monomer concentration. All curves correspond to experiments performed in triplicates.

### Fluorescence labelling of FoxP1

We prepared FoxP1 constructs in which the native cysteine residue (at position 61) was replaced by serine (C61S). Then, single-cysteine mutants S57C and V78C were generated to allow specific labelling. Before labelling, all buffers were sterile filtered and degassed. FoxP1 was concentrated to ~100 μM in buffer A (20 mM HEPES pH 7.8, 150 mM NaCl, 2 M GdmCl) supplemented with 0.5 mM Tris 2-carboxyethyl phosphine hydrochloride (TCEP). Then, 2.5 ml of concentrated protein were loaded onto a PD10 desalting column (GE Healthcare) and the protein was eluted with freshly degassed 3.5 ml of buffer A without TCEP. The eluted protein was immediately labelled with Oregon Green 488 (OG488) maleimide fluorophore (Thermo Fischer Scientific). Finally, SEC was performed to remove free fluorophore excess.

### Fluorescence measurements

Both intrinsic fluorescence and anisotropy measurements were performed in a Jasco FP-8300 spectrofluorometer (Jasco Corp, Japan), employing cells with 1 cm path lengths. In intrinsic fluorescence determinations, proteins were incubated in the specified buffer and spectra were recorded between 305 and 450 nm after excitation at 295 nm. The fluorescence intensity at 324 nm was used to monitor tertiary changes in both wt FoxP1 and H59A mutant. For anisotropy measurements, parallel and perpendicular fluorescent emission at 525 nm, after excitation at 495 nm, was recorded using labelled proteins. The G factor was determined using free OG488 dye at 1 μM in the specified buffer to calculate the observed anisotropy. No changes in G factor were observed upon changing the denaturant concentration (data not shown).

### Data analysis

Dissociation kinetics at specified pH values were fitted to a single exponential decay model, obtaining the observed dissociation rate (*k*_off_) and the equilibrium dimer fraction. Dimer fractions obtained in SEC experiments were used to determine the dissociation constant (K_d_) at the specified pH, as previously described^11^. Then, the association rates (*k*_on_) at each pH were determined using both K_d_ and *k*_off_ according to Equation 1:

1 $${{\rm{K}}}_{{\rm{d}}}=\frac{{k}_{{\rm{off}}}}{{k}_{{\rm{on}}}}$$

Dissociation rates were analysed in terms of Eyring equation^41^ to determine the free energy change between monomers or dimers and the transition state, ∆G^‡^_on_ and ∆G^‡^_off_ respectively.

Data from unfolding curves was fitted in terms of two-state (wt) or three-state mechanisms (H59A, S57C-OG488 and V78C-OG488) to obtain the free energy changes between the native (N), intermediate (I) and unfolded (U) states^11^.

The dependence of the resulting data on pH was determined using the Wyman-Tanford relationship^42^ (Equation 2):

2 $$\Delta {\rm{G}}=-\,{\rm{R}}{\rm{T}}\,\sum _{i}{\rm{l}}{\rm{n}}\{\frac{[(1+{10}^{({{\rm{p}}{\rm{K}}{\rm{a}}}_{i}^{{\rm{A}}}-{\rm{p}}{\rm{H}})})\cdot (1+{10}^{({{\rm{p}}{\rm{K}}{\rm{a}}}_{i}^{{\rm{B}}}-{{\rm{p}}{\rm{H}}}_{{\rm{r}}{\rm{e}}{\rm{f}}})})]}{[(1+{10}^{({{\rm{p}}{\rm{K}}{\rm{a}}}_{i}^{{\rm{B}}}-{\rm{p}}{\rm{H}})})\cdot (1+{10}^{({{\rm{p}}{\rm{K}}{\rm{a}}}_{i}^{{\rm{A}}}-{{\rm{p}}{\rm{H}}}_{{\rm{r}}{\rm{e}}{\rm{f}}})})]}\}$$

where R is the gas constant (in kcal·mol^−1^·K^−1^), T is temperature (310 K), ${{\rm{pKa}}}_{i}^{{\rm{A}}}$ and ${{\rm{pKa}}}_{i}^{{\rm{B}}}$ represent the pKa value of residue *i* in states A and B, and pH_ref_ corresponds to 5.0.

For the dissociation analysis, ${{\rm{pKa}}}_{i}^{{\rm{A}}}$ represents the pKa value of residue *i* in either the dimer or monomer states, whereas ${{\rm{pKa}}}_{i}^{{\rm{B}}}$ is the pKa value of residue *i* in the transition state (‡). For the folding analysis, ${{\rm{pKa}}}_{i}^{{\rm{A}}}$ represents the pKa value of *i* residue in the I state, and ${{\rm{pKa}}}_{i}^{{\rm{B}}}$ is the pKa value of *i* residue in the U state. In the case of mutant H59A, only one residue (H54) was assumed to be sensitive to pH changes. To obtain pKa values for H59 from Eq. 2, data from H59A was used as fixed parameters to obtain the second residue (H59) in the wt protein.

Fitting procedures were performed using the software GraphPad Prism 6.0c (www.graphpad.com).

### Molecular dynamics simulations

All-atom molecular dynamics (MD) were performed in NAMD 2.9^43^ using the CHARMM36m force field^44^. Initial models were taken from the monomeric structure of the A39P/C61Y mutant of human FoxP1 (PDB ID 2KIU)^8^. Mutated residues were reversed to the ones in the wt protein using VMD^45^. Two systems were prepared with this structure, one with neutral (HSD) and another with protonated (HSP) H59. Proteins were solvated in TIP3P water boxes with periodic boundary conditions, and charges were neutralized by the addition of Cl^−^ counter ions using VMD. Systems were minimized for 4,000 steps using the conjugated gradient algorithm. After minimization, proteins were slowly heated from 0 to 300 K and the whole system was equilibrated in the NPT ensemble for 20 ns. Three independent production MD simulations for each system were performed for 40 ns, using a time step of 2 fs. Inter-residue distance and hydrogen bond analysis were performed in VMD.

### Folding simulations

Coarse-grained structure-based models (SBM) of monomeric FoxP1 were generated using the SMOG web server^27^ using the default parameters. In these models each residue is represented as a single bead centred at its corresponding Cα atom. Bonded interactions are maintained by harmonic restraints, whereas only non-bonded residues separated in sequence by at least 3 residues and in contact in the native state (i.e. the distance between any pair of atoms from these residues is less than 6 Å) are given attractive Lennard-Jones interactions^46^ (Equation 3):

3 $${{\rm{V}}}_{{\rm{S}}{\rm{B}}{\rm{M}}}=\sum _{i < j-3}{\varepsilon }_{{\rm{C}}}[5{(\frac{{\sigma }_{ij}}{{r}_{ij}})}^{12}-6{(\frac{{\sigma }_{ij}}{{r}_{ij}})}^{10}]+\sum _{ij\notin {\rm{c}}{\rm{o}}{\rm{n}}{\rm{t}}{\rm{a}}{\rm{c}}{\rm{t}}{\rm{s}}}{\varepsilon }_{{\rm{N}}{\rm{C}}}{(\frac{{\sigma }_{ij}}{{r}_{ij}})}^{12}$$

where *ε*_C_ and *ε*_NC_ are the energies for native and non-native contacts, *σ*_*ij*_ is the distance between residue pairs in the native state and *r*_*ij*_ is their distance during the simulation.

To account for charge effects, these SBM models were supplemented with non-native charge interactions via a Debye-Hückel (DH) potential (Equation 4) as in previous works^26^:

4 $${{\rm{V}}}_{{\rm{D}}{\rm{H}}}={k}_{{\rm{e}}{\rm{l}}{\rm{e}}{\rm{c}}}B({\lambda }_{D})\sum _{i,j}\frac{{q}_{i}{q}_{j}{e}^{(-{r}_{ij}/{\lambda }_{D})}}{\varepsilon {r}_{ij}}$$

where *k*_elec_ is the Debye constant, *λ*_D_ is the Debye length that depends on the salt concentration (here, we used 0.05 M of monovalent salt, thus *λ*_D_ is 0.735^47^), *B*(*λ*_D_) is the Debye coefficient of the solution (corresponding to ~1 for dilute solutions^47^) and *ε* is the dielectric constant (here, 80). Charges *q*_*i*_ and *q*_*j*_ were placed on the Cα atoms corresponding to Asp, Glu (−1), Arg and Lys (+1), whereas H59 was either treated as neutral or protonated, in which case a positive charge was added.

Simulations at 30 different temperatures around the folding temperature T_F_, corresponding to the protein being always folded to always unfolded, were performed on GROMACS 4.5.4^48^ for 10^7^ steps using a time step τ of 0.0005. The weighted histogram analysis method^49^ was used to combine the simulation data from different temperatures into single free-energy profiles as a function of the global fraction of native contacts (Q). For this reaction coordinate, two residues are considered in contact if their distance is within 1.2 times the native distance. Also, the probability of a native contact being formed, *P*_*(i,j)*_, was calculated from simulations at T = T_F_.

## Supplementary information

Supplementary Information

## Data Availability

All experimental and computational data is available from the corresponding authors on request.
