# Author Correction: The protonation state of an evolutionarily conserved histidine modulates domain swapping stability of FoxP1

**DOI:** 10.1038/s41598-020-70337-y

**Published:** 2020-09-24

**Authors:** Exequiel Medina, Pablo Villalobos, Ricardo Coñuecar, César A. Ramírez-Sarmiento, Jorge Babul

**Affiliations:** 1 Departamento de Biología, Facultad de Ciencias, Universidad de Chile, Las Palmeras 3425, Casilla 653, Santiago, 7800003 Chile; 2 Institute for Biological and Medical Engineering, Schools of Engineering, Medicine and Biological Sciences, Pontificia Universidad Católica de Chile, Av. Vicuña Mackenna 4860, Santiago, 7820436 Chile

Correction to: *Scientific reports*, 10.1038/s41598-019-41819-5 published online 01 April 2019

The original version of this Article contained an error in the title of the paper, where the words “domain swapping” were incorrectly presented without a space between the words, as “domainswapping”. This has now been corrected in the PDF and HTML versions of the Article and in the accompanying Supplementary Information file.

