# Supplementary material for: The protonation state of an evolutionarily conserved histidine modulates domain swapping stability of FoxP1
Source: Sci Rep. 2019 Apr 1;9:5441. doi: 10.1038/s41598-019-41819-5 (PMC6443806; doi:10.1038/s41598-019-41819-5)
Supplement: Supplementary file 1 — Supplementary Information [file 41598_2019_41819_MOESM1_ESM.pdf]

SUPPLEMENTARY INFORMATION

The protonation state of an evolutionarily conserved histidine modulates domain swapping stability of FoxP1

Exequiel Medina[1], Pablo Villalobos[1], Ricardo Coñuecar[1], César A. Ramírez-Sarmiento[2*] and Jorge Babul[1*]

[1]Departamento de Biología, Facultad de Ciencias, Universidad de Chile, Las Palmeras 3425, Casilla 653, Santiago 7800003, Chile

[2]Institute for Biological and Medical Engineering, Schools of Engineering, Medicine and Biological Sciences, Pontificia Universidad Católica de Chile, Av. Vicuña Mackenna 4860, Santiago 7820436, Chile

[*]Correspondence:

César A. Ramírez-Sarmiento (cesar.ramirez@uc.cl); Institute for Biological and Medical Engineering, Schools of Engineering, Medicine and Biological Sciences, Pontificia Universidad Católica de Chile, Av. Vicuña Mackenna 4860, Santiago 7820436, Chile

Jorge Babul (jbabul@uchile.cl); Departamento de Biología, Facultad de Ciencias, Universidad de Chile, Las Palmeras 3425, Casilla 653, Santiago 7800003, Chile

The protonation state of an evolutionarily conserved histidine modulates domain swapping stability of FoxP1

Exequiel Medina, Pablo Villalobos, Ricardo Coñuecar, César A. Ramírez–Sarmiento and Jorge Babul

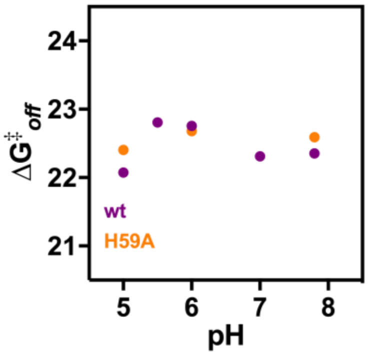

Figure S1. Kinetic properties of FoxP1 around the pKa of its H59 side chain. Comparison of $\Delta G^{\ddagger}_{off}$ (dissociation) of wt (purple) and H59A mutant (orange) as a function of pH. Values were calculated using the Eyring equation.

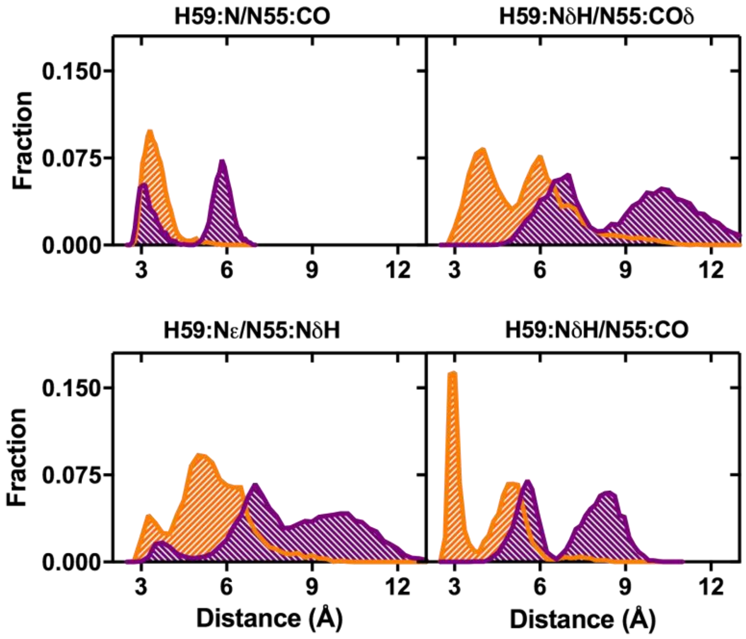

Figure S2. Distance distribution patterns obtained from simulations between backbone (N) and side–chain (N∂ and Nε) N atoms of H59 with carbonyl backbone (CO) and side chain (CO∂ and N∂H) of N55. All–atom molecular simulations were performed using neutral (HSD) and protonated (HSP) states of H59, and distances between selected atoms during the MD simulations were measured using VMD.

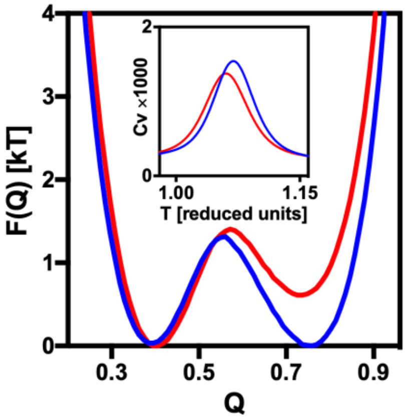

Figure S3. Changes in the folding landscape of FoxP1 upon protonation of H59. Free energy profile as a function of the fraction of native contacts (Q) for wt FoxP1 with neutral (blue) or protonated (red) H59 at the T_F (~1.058 reduced units) of neutral H59. Simulations were ran using a structure-based coarse-grained model augmented with electrostatic interactions. The inset shows the shift in the peak and area of the heat capacity curve upon protonation of H59.

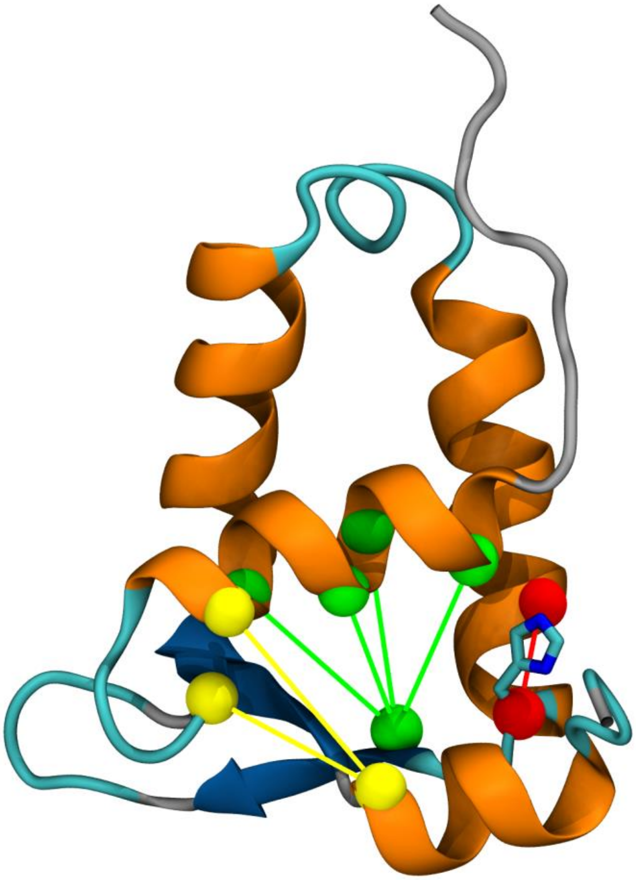

Figure S4. Mapping of native contacts stabilized in the folding transition state of FoxP1 upon deprotonation of H59. Cartoon representation of the monomer of FoxP1, with the residues involved in native contacts shown as spheres and residue H59 shown as sticks. Contacts between helix *H*1 and strand *S*2 are shown in green; contacts between helix *H*5, helix *H*1 and strand *S*2 are shown in yellow; and the native contact between residues H59 (helix *H*3) and N55 is shown in red.
